# Urinary Organophosphate Metabolites and Metabolic Biomarkers of Conventional and Organic Farmers in Thailand

**DOI:** 10.3390/toxics9120335

**Published:** 2021-12-04

**Authors:** Pornpimol Kongtip, Noppanun Nankongnab, Nichcha Kallayanatham, Jutamanee Chungcharoen, Chanapa Bumrungchai, Sumate Pengpumkiat, Susan Woskie

**Affiliations:** 1 Department of Occupational Health and Safety, Faculty of Public Health, Mahidol University, 420/1 Rajvidhi Road, Bangkok 10400, Thailand; noppanun.nan@mahidol.ac.th (N.N.); nichcha.kal@gmail.com (N.K.); yiesipttt@gmail.com (J.C.); chanapa.b23@gmail.com (C.B.); sumate.pen@mahidol.ac.th (S.P.); 2 Center of Excellence on Environmental Health and Toxicology, EHT, Bangkok 10400, Thailand; 3 Department of Public Health, University of Massachusetts Lowell, One University Ave, Lowell, MA 01854-2867, USA; Susan_Woskie@uml.edu

**Keywords:** organophosphate metabolites, metabolic biomarkers, conventional farmers, organic farmers

## Abstract

Organophosphate (OP) pesticides are used by most farmers to remove insects and to increase productivity; however, questions remain on the long-term health impacts of their use. This study assessed the relationship between OP biomarker levels and metabolic biomarker parameters. Conventional farmers (*n* = 213) and organic farmers (*n* = 225) were recruited, interviewed, and had physical health examinations. Serum glucose and lipid profiles, triglycerides, total cholesterol, high-density lipoproteins (HDL), and low-density lipoproteins (LDL) were measured. The average age, gender, education, and self-reported agricultural work time, work in second jobs, smoking status, alcohol consumption, insecticide use at home, home location near farmlands and years of pesticide use were significantly different between the conventional and organic farmers. The urinary OP metabolite levels were also significantly different between the two groups. With an increase in urinary diethyl phosphate, dimethyl phosphate and dialkyl phosphate metabolites, the total cholesterol, LDL and HDL, were significantly increased for all farmers after controlling for age, gender, alcohol consumption, years of pesticide use, and home location near farmlands. The results are consistent with our previous studies which suggests that pesticide usage, especially organophosphates, may increase the risk of cardiovascular disease and stroke among Thai farmers.

## 1. Introduction

Thailand pesticide imports were reduced from 131,148 tons in 2019 to 98,254 tons in 2020, which is probably related to the ban implemented in Thailand in June 2020 for the organophosphate chlorpyrifos and the herbicide paraquat, along with limitations on the use of the herbicide glyphosate [1]. Pesticides are widely applied to prevent or treat infestations of weeds, insects, or fungi. Most farmers select types of pesticides to use based on the recommendations of the local pesticide shop and do not have knowledge on their proper use [2,3]. The most used insecticides in Thailand are the organophosphates and synthetic pyrethroids [4]. Conventional farmers sometimes mix and spray several pesticides together to save time and the cost of spraying, if sprayers are hired [5]. There were 805 pesticide poisoning cases from organophosphate and carbamate pesticides and 58 deaths reported for the period of October 2018 to June 2019 in Thailand [6].

The National Plan for Organic Farming in Thailand aims to increase organic farming to a total of 1.3 million rai by 2021 and reserve 40% of all organic produce for domestic consumption [7]. Organic farmers must use certified organic farming practices for all their crops, including rice, vegetables, and fruits. Previously, we reported results from both a cross-sectional and longitudinal study of conventional and organic farmers where we found that cross-sectionally, conventional farmers had a significantly higher risk than organic farmers of abnormal body mass index (BMI), waist circumference, body fat %, triglyceride, total cholesterol, and low-density lipoproteins (LDLs), even after accounting for potential confounders [8]. In our longitudinal study, the conventional farmers had significantly higher BMI, waist circumference, total cholesterol, LDL, high-density lipoproteins (HDLs), blood glucose, systolic and diastolic blood pressure than organic farmers [9]. This study aimed to assess the association between organophosphate pesticide metabolite levels measured in conventional and organic farmers, and their metabolic biomarker levels.

## 2. Materials and Methods

### 2.1. Study Population and Data Collection

To ensure a variety of farmers growing different types of plants, we selected sugarcane farmers in Nakhonsawan province, rice and vegetable farmers in Phitsanulok province, and rice, fruit and vegetable organic farmers in Yasothon province. Help in recruiting was received from the health-promoting hospitals/primary care units (HPH/PCUs) in the areas, community leaders, and public health volunteers. The study recruited male and female farmers over 18 years of age who were free of a current diagnosis of diabetes, high blood pressure, thyroid, or heart disease. We selected only one farmer per family, focusing on a member who mainly worked in the agricultural fields. For conventional farmers, the farmer had to spray pesticides on their own or rented farms. For organic farmers, all crops had to be certified organic. At recruitment we enrolled 243 conventional farmers and 235 organic farmers [8]. After 8 months, we arranged for a physical health checkup for study subjects resulting in 213 conventional farmers and 225 organic farmers. This was a loss to follow-up of 8.23% for the conventional farmers and 4.26% for the organic farmers. We hired site officers to interview the subjects at their homes using a questionnaire consisting of screening questions, farmer characteristics, health behaviors, family health history, food consumption behaviors, home and demographic information, self-reported health problems, agricultural activities, history of pesticide use, and mental health stress questionnaire [8,10].

On the day before the sample collection all farmers were given a 50-mL polyethylene container to collect their first morning void urine and were instructed to collect urine as soon as they woke up. They brought their urine samples to the health promoting hospital on the morning (7.00–9.00 a.m.) of their physical health examinations. Data on weight, height, waist circumference, body composition (Tanita model DC-360, Amsterdam, Netherland), blood pressure (taken twice, 10 min apart and averaged), and blood samples were collected during the physical examination. Sera extracted from blood samples in non-heparinized vacutainer tubes was stored at −20 °C before analysis. The urine samples were analyzed for organophosphate metabolites. Serum was analyzed for glucose, triglycerides, total cholesterol, HDL, and LDL by an AU5800 apparatus (Beckmann Coulter, Atlanta, GA, USA) at Buddhachinaraj Hospital, the regional medical center in the Phitsanulok province, using standard clinical laboratory methods. 

All subjects provided their written informed consent before they participated in the study. The study protocol was approved 28 August 2015 by the Ethics Committee of Human Research, Faculty of Public Health, Mahidol University (MUPH 2015-146).

### 2.2. Analysis of Organophosphate Metabolites in Urine

The analysis of the non-specific organophosphate (OP) metabolites of dimethyl phosphate (DMP), dimethyl thiophosphate (DMTP), dimethyl dithiophosphate (DMDTP), diethyl phosphate (DEP), diethyl thiophosphate (DETP), and diethyl dithiophosphate (DEDTP) followed the GC-MS method of Prapamontol et al. [11] except that the derivatization with 2,3,4,5,6-Pentafluorobenzyl bromide was conducted at 80 °C for 1.5 h. The calibration curve of the 6 OP metabolites ranged from 10 to 1000 ng/mL. The accuracy and precision of the OPs at 50 and 150 ng/mL ranged from 96.8 to 117.3% with a relative standard deviation (RSD) of less than 7%, except for DMP where the RSD was 17%. The detection limit of the OP metabolites in urine was 0.57 ng/mL for DMP, 1.17 ng/mL for DMTP, 1.06 ng/mL for DMDTP, 0.54 ng/mL for DEP, 0.88 ng/mL for DETP, and 1.52 ng/mL for DEDTP. For concentrations below the detection limit, we substituted the detection limit divided by the √2 [12]. The creatinine in urine was analyzed (mg/dL) using an enzymatic method with a linear concentration range of 1–500 mg/dL and a detection limit of 0.16 mg/dL [13]. All urinary metabolites were creatinine corrected to units of ng/g creatinine and converted to nmoles/g creatinine for presentation. The combined results were presented as ΣDMP = DMP + DMTP + DMDTP, ΣDEP = DEP + DETP + DEDTP, and DAP = ΣDMP + ΣDEP.

### 2.3. Statistical Analysis

All statistical analyses were performed by SPSS for Windows, version 23 (IBM Thailand Co., Ltd., Bangkok, Thailand). Descriptive analyses of the demographic characteristics were performed using chi-square test, Fisher’s exact test and independent *t*-tests. Due to the skewed distribution of urinary OP metabolites, the natural logarithm was used in all analyses. The natural log values for the urinary OP metabolite concentrations were compared between conventional and organic farmers using an independent *t*-test. We used a generalized linear model to investigate the effect of a one unit increase in ΣDMP, ΣDEP, and DAP on the RR of metabolic biomarker outcomes in univariate analyses. To develop the models examining the impact of DAP, ΣDEP, and ΣDMP on metabolic biomarker levels we first conducted a series of univariate analyses with each metabolic outcome (BMI, waist circumference, %Body Fat, blood pressure, and blood glucose levels, as well as triglyceride and lipid levels (HDL, LDL, total cholesterol)) and each of the potential predictors listed in Table 1. We found that that age, gender, having a second job, drinking alcohol, insecticide use at home, living near a farm and years of pesticide use were significant predictors of several health outcomes. A Spearman rho correlation was used to test for the collinearity of the significant exposure related variables: insecticide use at home, living near a farm, second job and year of pesticide use; they were all significantly correlated at *p* < 0.001. We also tested for the collinearity effect in the linear regression model to select the parameters that were important according to the literature review and showed lower collinearity (VIF). Therefore, we selected only two parameters, living near a farm and year of pesticide use to include in the models along with age, gender, and drinking alcohol.

## 3. Results

### 3.1. Characteristics of Conventional and Organic Farmers

The average age of organic farmers (50.2 years) was significantly higher than that of conventional farmers (53.2 years) (Table 1). The conventional farmers had significantly higher fraction of male farmers (74.2%) compared with female farmers (25.8%). The organic farmers had significantly higher education levels than those conventional farmers. The hours of work for organic farmers were significantly higher than those of conventional farmers, and they were also more likely to have second jobs. The conventional farmers were significantly more likely to drink alcohol and smoke cigarettes than the organic farmers. Conventional farmers were more likely to live near their farm (84.5% vs. 46.64%), use pesticides at home (89.7% vs. 14.7%), and had higher years of pesticide use (26.71 vs. 16.25 years) than the organic farmers.

### 3.2. Comparison of Metabolic Biomarkers between Conventional and Organic Farmers

The conventional farmers had significantly higher metabolic biomarker levels than organic farmers for BMI, waist circumference, triglyceride, total cholesterol, LDL, HDL, blood glucose, and diastolic blood pressure (Table 2). The % body fat and systolic blood pressure were not significantly different between the two groups.

### 3.3. Comparison between Urinary OP Metabolites between Conventional and Organic Farmers

The percentage of urinary OP metabolites with detectable levels was highest for DETP (64.8%) followed by DEDTP (62.9%), DEP (60.1%), DMP (51.2%), DMTP (49.3%), and DMDTP (2.8%) for conventional farmers (Table 3).

The detection frequency for urinary OP metabolites among organic farmers was considerably lower than those of conventional farmers. For organic farmers, the detection frequency was highest for DETP (61.3%) followed by DEP (12.9%), DMTP (12.4%), DEDTP (10.2%), and DMP (8.4%). In addition, the conventional farmers had significantly higher urinary OP metabolite levels than organic farmers for all metabolites except DMDTP, as well as for the combined categories of OP metabolites: ΣDMP, ΣDEP, and DAP.

### 3.4. Comparison of Urinary OP Metabolites of This Study with Other Studies

Since most of the urinary OP levels presented in the literature used μg/g creatinine, we calculated concentrations in the same units for comparison. The GM of urinary DAP metabolites (87.64 μg/g creatinine) in conventional farmers in this study was higher than most studies, except for one study from Korea (287.10 μg/g creatinine) [14] (Table 4). However, the maximum DAP level (2186.38 μg/g creatinine) in this current study was lower than the study of Choi et al. [14] of 5198.60 μg/g creatinine and Panuwet et al. [15] of 6476 μg/g creatinine.

### 3.5. Model for Change in Metabolic Biomarkers per Unit of OP Metabolite

An increase in one nmole/g creatinine of DAP, ΣDEP, and ΣDMP, significantly in-creased the metabolic biomarkers of total cholesterol, LDL, and HDL after controlling for age, gender, alcohol consumption, year of pesticides used and living near farm (Table 5, Table 6 and Table 7). The BMI, waist circumference, % body fat, triglyceride, blood glucose, systolic and diastolic blood pressure were not significantly increased with the increase in one nmole/g creatinine of DAP, ΣDEP, and ΣDMP.

## 4. Discussion

The average age of organic farmers was significantly higher than those of conventional farmers because most of the organic farmers in the current study used to work with pesticides before changing to organic farming. Chouichom and Yamao [18] reported that longer farm experience (higher age) and higher education were supporting factors in the change into an organic farming system. Likewise, a study among smallholder farmers in Uganda found that the average age of organic farmers (53.5 years) was higher than those conventional farmers (45 years), but in Costa Rica the average of organic farmers (30 years) was lower than those conventional farmers (35 years). In Costa Rica, organic and sustainable agriculture are considered better choices for export; therefore, younger farmers tend to choose organic farming [19,20]. On the other hand, organic and sustainable agriculture is perceived as an “old men’s farming practice” in Uganda [21].

Organic farmers’ working hours were significantly higher than those of conventional farmers, and organic farmers were significantly more likely to have a second job than conventional farmers. Conventional farmers are more likely to use machinery in farming than organic farmers [22]. The conventional farmers were more likely to be male than female because our inclusion criteria stated that conventional farmers had to spray pesticides on their farms. Usually, male farmers are the persons who spray pesticides using backpack spraying equipment which weighs approximately 10–20 kg. This study found conventional farmers were more likely to be smokers and drinkers than the organic farmers. This is probably because conventional farmers are more likely to be male, and alcohol consumption and smoking are more likely to occur in males than females in Thailand [23,24].

The results showed that conventional farmers had significantly higher BMI, waist circumference, triglyceride, total cholesterol, LDL, HDL, blood glucose and diastolic blood pressure than those organic farmers. This was similar to our previous longitudinal study, which reported that conventional farmers had significantly higher total cholesterol, LDL, HDL, blood glucose, systolic and diastolic blood pressure, BMI, and waist circumference than organic farmers [9]. The current result was also similar to the study of Pothu et al. [25] which reported that patients with decreased cholinesterase levels (higher OP exposure) had significantly higher serum cholesterol, triglycerides and LDL levels than those with normal cholinesterase levels (lower OP exposure).

The urinary OP metabolite levels among conventional farmers were significantly higher than those of organic farmers. Our conventional farmers sprayed pesticides on their own farm or they were hired to spray on other people’s farms. These conventional farmers sprayed insecticide most commonly 2–4 times per crop (49.3% reported), while 11.3% reported spraying once per month, 26.1% reported spraying two to four times per month, 10.3% reported spraying once per week, and 3.0% reported spraying five to seven times per week. Organophosphate pesticide use reported by conventional farmers were chlorpyrifos 26.6%, phenthoate 1%, EPN 2.5%, diazinon 2%, dichlovos 0.5%, dichrotophos 0.5%, methamidophos 0.5%, monochrotophos 0.5%, prophenophos 5%, acephate 1%, methyl parathion 0.5% and triazophos 1.5%. For the DEP metabolite group in this study, 28.6% reported using chlorpyrifos and diazinon while for the DMP metabolite group, 1.5% reported using dichlovos, dichrotophos, and methyl parathion [26,27].

The concentrations of urinary OP metabolites found for organic farmers can come from pesticides used in the home because these organic farmers were not allowed to use any chemicals on their farms. However, some of these organic farmers reported use of pesticides at home. Many organic farmers (47%) live within 1 km of farms and some of those farms may use pesticides resulting in potential pesticide drift exposures. Organic farmers can also be exposed to OP pesticides through purchased prepared or raw food products that contain OP residues. Since conventional and organic farmers in this study did not have a significant difference in their self-reported frequency of eating vegetables and fruits in the past month [8], the conventional farmers may also have some of their metabolite levels due to food OP residues. In addition to their exposures from the mixing and spraying of pesticides and their work in fields sprayed with pesticides, conventional farmers may also have home exposures from pesticide drift, since 84.5% of them living near farmlands.

Comparing the urinary OP metabolites with other studies, the reason the Korean study by Choi et al. [14] had the highest urinary GM for DAP is most likely because 87.7% of the subjects sprayed pesticides and 12% assisted in spraying. Forty-five percent of these subjects had sprayed pesticides less than four days before the urine sample collection, increasing the likelihood of high urinary DAP levels. Motsoeneng et al. [17] of South Africa also reported higher median DAP levels than this study. Although no information on pesticide spraying was provided, it is likely that the urinary OP metabolite levels of farm workers in that study were high because they reported re-entry into field on the same day (67%) or 1 to 7 days (27%) after pesticide spraying. This study detected a higher GM of DAP than two other studies in the northern part of Thailand [15,16]. In Sapbamrer et al. [16], 57.1% of farmers sprayed pesticides once a month and the rest did not spray pesticides, compared with this study where 65.5% reported spraying once a month or more often. The Panuwet et al. [15] study did not have information about pesticide spraying but the women on the farms were classified as farm workers even though only 2% of them reported being pesticide applicators, most likely explaining the much lower DAP levels than in our study.

We previously found that conventional farmers had significantly higher abnormal metabolic biomarkers than organic farmers, including BMI, waist circumference, % body fat, triglyceride, total cholesterol, LDL, and HDL levels, even after controlling for other covariates [8]. In Kongtip et al. [9] we found that 10 days of insecticide spraying significantly increased metabolic biomarkers of total cholesterol, LDL, and HDL even after controlling for other covariates. In this study, when we modeled the impact of urinary OP metabolites, the total cholesterol, LDL, and HDL significantly increased with an increase in urinary ΣDEP, ΣDMP, and DAP metabolites. Leonel Javeres et al. [28] showed significantly increased LDL, lipoparticles and decreased HDL among chronically OP exposed residents of cotton farming areas in Cameroon and Pakistan.

In animal studies, rats exposed to OP pesticides had significantly higher HDL compared with the controls [29,30] Studies show that OP insecticides generally increase total cholesterol and total lipid levels; chlorpyrifos (DEP metabolite) was found to elevate total cholesterol levels in rats [31], diazinon (DEP metabolite) was also found to elevate total cholesterol in rats [32], and dichlovos (DMP metabolite) was shown to increase total cholesterol but decrease the LDL in rats [33]. A meta-analysis of the impact of diazinon OP in rodents and fish species showed that diazinon significantly increased total cholesterol, triglycerides, LDL, and significantly decreased HDL in the exposed group compared with the control group [34].

Increased serum cholesterol levels may be a sign of liver damage [31]. Leonel Javeres et al. [28] found dysregulated liver and kidney function profiles in chronically exposed residents of cotton farming areas [28]. Ranjbar et al. [35] reported that cardiometabolic health outcomes, blood pressure, cholesterol, LDL and HDL, differed depending on the specific OP metabolite being examined, with higher BMIs amplifying health risk.

Limitations of this study include the possibility that OP levels are a surrogate for differences in the way of life, diet, and work environment between the organic and conventional farmers that we were unable to control for in our models. The self-report of the type and frequency of insecticides used could be under reported because of recall bias. This is a cross-sectional study measuring only one spot urine sample of organophosphate pesticide metabolites from conventional and organic farmers; thus, exposure estimates may be biased.

## 5. Conclusions

With the increase in urinary ΣDEP, ΣDMP and DAP, metabolites, the metabolic biomarkers, total cholesterol, LDL, and HDL significantly increased among farmers. Further research is needed to explore the mechanisms by which OP pesticides alter metabolic biomarkers.

## Figures and Tables

**Table 1:** Characteristic and risk factors of conventional farmers (*n* = 213) and organic farmers (*n* = 225).

Variables	Conventional Farmers *n* (%)	Organic Farmers *n* (%)	*p*-Value
Age			
Min–max	18–69	28–79	
Mean (SD)	50.22 (11.1)	53.20 (10.3)	0.005 ^§^
Gender			
Male	158 (74.2)	115 (51.1)	<0.001 ^†^
Female	55 (25.8)	110 (48.9)	
Educational level			
Below elementary	14 (6.6)	4 (1.8)	0.035 ^†^
Elementary	122 (57.3)	125 (55.6)	
High school	72 (33.8)	85 (37.8)	
Bachelor or higher	5 (2.3)	11 (4.9)	
Marital status			
Single	21 (10.1)	13 (6)	0.032 ^†^
Married	179 (86.1)	185 (84.9)	
Widowed/divorced	8 (3.8)	20 (9.2)	
Agricultural work time (h/week)			
Mean (SD)	26.9 (13.8)	28.8 (17.2)	0.011 ^§^
Have second Job			
Yes	49 (23)	128 (56.9)	<0.001 ^†^
No	164 (77)	97 (43.1)	
Second job work time (h/week)			
Mean (SD)	24.9 (13.6)	26.6 (17.8)	0.048 ^§^
Alcohol intake			
Current drinker	136 (63.8)	91 (41)	<0.001 ^†^
Nondrinker	77 (36.2)	131 (59)	
Smoking			
Current smoker	59 (26.9)	36 (16.1)	0.006 ^†^
Nonsmoker	155 (73.1)	188 (83.9)	
Living near farms (within 1 km)			
Yes	180 (84.5)	104 (46.64)	<0.001 ^†^
No	33 (15.4)	119 (53.36)	
Insecticide use at home			
Yes	191 (89.7)	33 (14.7)	<0.001 ^†^
No	22 (10.3)	191 (85.3)	
Year of pesticide use (year)			
Min–Max	4–51	0–45	
Mean (SD)	26.71 (12.75)	16.25 (11.64)	<0.001 ^§^

† = chi-square test, ^§^ = independence *t*-test.

**Table 2:** Comparison of conventional (*n* = 213) and organic farmers (*n* = 225) for metabolic biomarkers using independent *t* test.

Health Outcomes	Conventional Farmers Mean (SD)	Organic Farmers Mean (SD)	*p-Value
BMI (kg/m^2^)	24.51 (4.73)	23.15 (3.58)	0.001
Waist circumference (cm)	83.00 (10.17)	79.72 (10.22)	0.001
% Body Fat (%)	27.04 (8.67)	26.78 (9.25)	0.763
Triglyceride (mg/dL)	170.59 (137.52)	144.07 (104.24)	0.024
Total cholesterol (mg/dL)	231.69 (42.29)	192.77 (56.21)	<0.001
HDL (mg/dL)	52.89 (11.39)	39.85 (12.93)	<0.001
LDL (mg/dL)	148.54 (38.99)	124.21 (42.18)	<0.001
Blood glucose (mg/dL)	109.36 (26.09)	102.56 (23.41)	0.004
Systolic BP (mmHg)	134.58 (17.35)	133.46 (17.09)	0.500
Diastolic BP (mmHg)	82.14 (11.72)	79.14 (9.31)	0.003

* = Significant at *p* < 0.05.

**Table 3:** Organophosphate metabolite comparison between conventional and organic farmers using independent *t* test on natural log of levels.

OP Metabolites	Conventional Farmers		Organic Farmers		*p*-Value
	% Detectible	GM (Range) (nmole/g Creatinine)	% Detectible	GM (Range) (nmole/g Creatinine)	
DMP	51.17	37.72 (0.73–6809.75)	8.40	3.87 (0.89–355.32)	<0.001
DMTP	49.30	41.06 (1.3–4674.27)	12.44	6.81 (0.94–278.27)	<0.001
DMDTP	2.82	4.68 (3.24–808.5)	0.0	4.45 (1.33–20.56)	0.518
DEP	60.10	29.63 (0.35–5800.85)	12.89	3.17 (0.3–178.11)	<0.001
DETP	64.79	50.83 (0.84–9343.99)	61.30	7.47 (0.17–86.26)	<0.001
DEDTP	62.91	22.17 (1.75–805.00)	10.22	2.92 (0.05–24.94)	<0.001
ΣDMP	–	183.20 (3.15–6819.10)	–	17.01 (3.87–637.05)	<0.001
ΣDEP	–	188.78 (4.00–12,829.68)	–	17.50 (2.71–268.35)	<0.001
DAP	–	581.67 (13.03–13,278.29)	–	37.83 (8.04–905.39)	<0.001

DMP: Dimethylphosphate, DMTP: Dimethylthiophosphate, DMDTP: Dimethyldithiophosphate. DEP: Diethylphosphate, DETP: Diethylthiophosphate, DEDTP: Diethyldithiophosphate. DAP: ΣDEP + ΣDMP, ΣDEP: DEP + DETP + DEDTP, ΣDMP: DMP + DMTP + DMDTP.

**Table 4:** Comparison of DAP (μg/gcreatinine) for different groups of farmers.

Reference	Country	Type of Farmers	*n*	DAP (μg/g Creatinine)	
				GM/Median	Max
This study	Thailand	Conventional farmers	213	87.64	2186.38
This study	Thailand	Organic farmers	225	5.92	127.7
Choi et al. [14]	Korea	Farmers	308	287.10	5198.60
Sapbamrer et al. [16]	Thailand	Farmers	84	6.43	163.90
Panuwet et al. [15]	Thailand	Farmers	136	34.8	6476
Motsoeneng et al. [17]	South Africa	Women on farm	101	Median 141.42	–

**Table 5:** Individual generalized linear model models for change in each metabolic biomarker per one unit of Ln ΣDMP. Adjusted models include covariates for age, gender, alcohol consumption, years of pesticide use, living within 1 km of a farm.

Health Outcome	All Farmers	*p-Value	All Farmers	*p-Value
	Crude RR (95%CI)		Adjusted RR (95%CI)	
BMI (kg/m^2^)	1.09 (088–1.34)	0.448	1.01 (0.78–1.31)	0.931
Waist circumference (cm)	1.14 (0.68–1.92)	0.610	0.92 (0.52–1.66)	0.792
% Body Fat (%)	0.95 (0.61–1.47)	0.803	1.07 (0.74–1.57)	0.716
Triglyceride (mg/dL)	0.18 (0.0–69.96)	0.576	0.01 (1.04 × 10^−5^–6.26)	0.156
Total cholesterol (mg/dL)	1102.61 (95.06–12,789.53)	<0.001*	827.98 (59.22–11,577.17)	<0.001 *
HDL (mg/dL)	8.84 (4.73–16.51)	<0.001*	6.70 (3.44–13.04)	<0.001 *
LDL (mg/dL)	197.94 (25.64–1527.75)	<0.001*	242.70 (27.27–2160.28)	<0.001 *
Blood glucose (mg/dL)	1.14 (0.42–3.09)	0.799	1.14 (0.42–3.09)	0.798
Systolic BP (mmHg)	0.81 (0.35–1.90)	0.629	0.81 (0.35–1.90)	0.628
Diastolic BP (mmHg)	0.85 (0.47–1.52)	0.583	0.85 (0.47–1.52)	0.581

* = Significant at *p* < 0.05.

**Table 6:** Individual generalized linear model models for change in each metabolic biomarker per one unit of Ln ΣDEP. Adjusted models include covariates for age, gender, alcohol consumption, years of pesticide use, living within 1 km of a farm.

Health Outcome	All Farmers	* *p*-Value	All Farmers	* *p*-Value
	Crude RR (95%CI)		Adjusted RR (95%CI)	
BMI (kg/m^2^)	1.26 (1.01–1.56)	0.038 *	1.20 (0.94–1.53)	0.153
Waist circumference (cm)	1.71 (1.01–2.91)	0.047 *	1.38 (0.74–2.59)	0.311
% Body Fat (%)	1.02 (0.63–1.64)	0.946	1.26 (0.86–1.84)	0.236
Triglyceride (mg/dL)	1.27 (0.00–405.20)	0.935	0.01 (5.2 × 10^−6^–33.74)	0.280
Total cholesterol (mg/dL)	221.30 (16.70–2933.28)	<0.001 *	57.00 (2.74–1185.93)	0.009 *
HDL (mg/dL)	8.19 (4.19–15.98)	<0.001 *	4.60 (2.13–9.96)	<0.001 *
LDL (mg/dL)	25.07 (2.77–226.80)	0.004 *	14.59 (1.22–174.51)	0.034 *
Blood glucose (mg/dL)	0.932 (0.28–3.08)	0.909	1.02 (0.37–2.85)	0.968
Systolic BP (mmHg)	0.92 (0.36–2.35)	0.861	1.02 (0.38–2.73)	0.969
Diastolic BP (mmHg)	1.27 (0.69–2.34)	0.447	1.31 (0.66–2.58)	0.441

* = Significant at *p* < 0.05.

**Table 7:** Individual generalized linear model models for change in each metabolic biomarker per one unit of Ln DAP. Adjusted models include covariates for age, gender, alcohol consumption, years of pesticide use, living within 1 km of a farm.

Health Outcome	All Farmers	* *p*-Value	All Farmers	* *p*-Value
	Crude RR (95%CI)		Adjusted RR (95%CI)	
BMI (kg/m^2^)	1.23 (1.00–1.51)	0.054	1.15 (0.89–1.48)	0.287
Waist circumference (cm)	1.58 (0.93–2.68)	0.093	1.21 (0.64–2.30)	0.552
% Body Fat (%)	1.01 (0.63–1.61)	0.977	1.26 (0.85–1.85)	0.250
Triglyceride (mg/dL)	1.07 (0.00–482.56)	0.983	0.01 (1.6 × 10^−6^–13.06)	0.184
Total cholesterol (mg/dL)	2217.93 (171.11–28,748.74)	<0.001 *	1134.08 (58.58–21,955.72)	<0.001 *
HDL (mg/dL)	12.88 (6.69–24.83)	<0.001 *	8.30 (3.88–17.77)	<0.001 *
LDL (mg/dL)	193.44 (24.49–1593.26)	<0.001 *	229.79 (21.72–2430.59)	<0.001 *
Blood glucose (mg/dL)	1.38 (0.50–3.80)	0.534	1.02 (0.36–2.92)	0.973
Systolic BP (mmHg)	0.94 (0.38–2.32)	0.892	0.99 (0.38–2.53)	0.976
Diastolic BP (mmHg)	1.16 (0.65–2.08)	0.619	1.13 (0.58–2.18)	0.724

* = Significant at *p* < 0.05.

## Data Availability

The data presented in this study are available on request from the corresponding author. The data are not publicly available due to privacy of subjects.
